# Visual Neglect after PICA Stroke—A Case Study

**DOI:** 10.3390/brainsci12020290

**Published:** 2022-02-19

**Authors:** Nora Geiser, Brigitte Charlotte Kaufmann, Henrik Rühe, Noortje Maaijwee, Tobias Nef, Dario Cazzoli, Thomas Nyffeler

**Affiliations:** 1 Neurocenter, Luzerner Kantonsspital, 6000 Lucerne, Switzerland; nora.geiser@luks.ch (N.G.); brigitte.kaufmann@luks.ch (B.C.K.); henrik.ruehe@luks.ch (H.R.); noortje.maaijwee@luks.ch (N.M.); dario.cazzoli@luks.ch (D.C.); 2 ARTORG Center for Biomedical Engineering Research, University of Bern, 3008 Bern, Switzerland; tobias.nef@unibe.ch; 3 Department of Neurology, Inselspital, University Hospital, University of Bern, 3010 Bern, Switzerland; 4 Institut du Cerveau—Paris Brain Institute—ICM, Inserm, CNRS, Sorbonne Université, 75013 Paris, France; 5 Department of Psychology, University of Bern, 3012 Bern, Switzerland

**Keywords:** neglect, PICA, video-oculography, free visual exploration, stroke

## Abstract

After cerebellar stroke, cognition can be impaired, as described within the framework of the so-called Cerebellar Cognitive Affective Syndrome (CCAS). However, it remains unclear whether visual neglect can also be part of CCAS. We describe the case of a patient with a subacute cerebellar stroke after thrombosis of the left posterior inferior cerebellar artery (PICA), who showed a left-sided visual neglect, indicating that the cerebellum also has a modulatory function on visual attention. The neglect, however, was mild and only detectable when using the sensitive neuro-psychological Five-Point Test as well as video-oculography assessment, yet remained unnoticed when evaluated with common neglect-specific paper-pencil tests. Three weeks later, follow-up assessments revealed an amelioration of neglect symptoms. Therefore, these findings suggest that visual neglect may be a part of CCAS, but that the choice of neglect assessments and the time delay since stroke onset may be crucial. Although the exact underlying pathophysiological mechanisms remain unclear, we propose cerebellar–cerebral diaschisis as a possible explanation of why neglect can occur on the ipsilateral side. Further research applying sensitive assessment tools at different post-stroke stages is needed to investigate the incidence, lesion correlates, and pathophysiology of neglect after cerebellar lesions.

## 1. Introduction

The Cerebellar Cognitive Affective Syndrome (CCAS, also referred to as Schmahmann Syndrome [1]) demonstrates that the cerebellum is not only involved in the control of movement, but also cognition. For instance, cerebellar lesions—in particular strokes in the territory of the posterior inferior cerebellar artery (PICA; [2])—may not only lead to impairments in executive functions, changes in personality and language difficulties, but also to impairments in spatial cognition [1,3,4,5,6,7]. However, visual neglect—a very common form of spatial attentional impairment—has been rarely reported after cerebellar stroke, and findings remain ambiguous. Sparse case reports suggest that neglect might occur after cerebellar stroke [8,9]. On the other hand, some larger-scale studies failed to demonstrate an involvement of the cerebellum in spatial attention allocation [10,11,12]. 

Here, we present the case of a patient with a left subacute stroke in the territory of the PICA leading to left-sided visual neglect. Crucially, the neglect was transient and only apparent when using sensitive neuropsychological and oculomotor assessments, and would have gone unnoticed if only common neuropsychological paper-pencil tests had been administered. This suggests that neglect after cerebellar stroke is probably more common than previously assumed, and its detection requires early assessments with sensitive measures.

## 2. Materials and Methods

**Figure 1:**
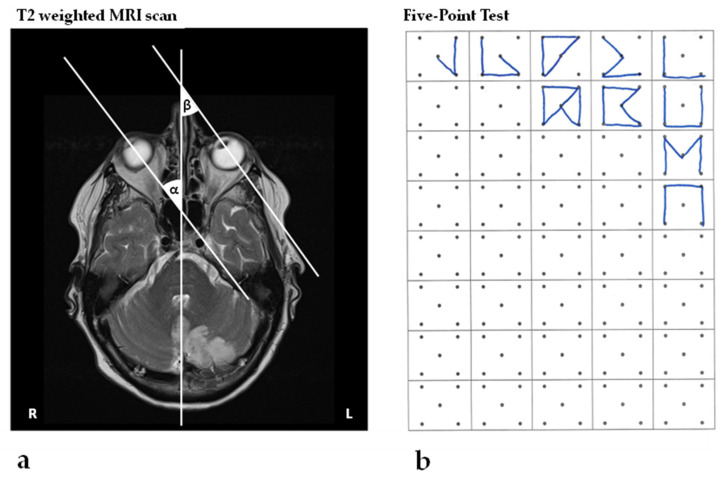
MRI and the Five-Point Test. (**a**) T2–weighted contrast MRI images showing a stroke in the left PICA territory one day post-stroke. The neuroimaging of the eye position, by means of the angles formed by the intersection of the ocular axes of the right (α) and left (β) eye and the midline structures of the head, is indicative of spatial neglect, as described by Becker and Karnath [29]; (**b**) shows the patient’s performance in the Five-Point Test, revealing a significant rightward shift of the spatial distribution of drawn designs 24 days post-stroke (CoC = 0.338; see for example [17]).

We present the case of a 79 year-old female patient with a subacute stroke within the territory of the left PICA (Figure 1a). In medical reports from the external stroke unit, mild ataxia in the left upper and lower limbs as well as a gait ataxia were described. There were no signs of neglect or nystagmus. One day post-stroke, an MRI was performed which showed an acute stroke within the territory of the terminal branch of the left PICA; there were no other concomitant infra- or supratentorial lesions.

Twenty-four days after stroke, several conventional, neglect-specific paper-pencil tests such as the Bells Test [13], the Sensitive Neglect Test (SNT) [14] and the Line Bisection Test [15] were performed as part of the clinical routine in our inpatient neurorehabilitation unit. In addition to the above-mentioned neglect-specific assessments, the Five-Point Test [16] was conducted. In the Five-Point Test, patients actively generate designs instead of cancelling targets—this assessment is therefore known to be more demanding and shows a higher sensitivity compared to cancelling tests and the Line Bisection Test [17]. To evaluate if a patient scored within a normal range, the Center of Cancellation (CoC) was calculated for both the cancellation and the Five-Point Test. The CoC is a measure used to define the centre of mass of the spatial distribution of detected items [18]. In case of an unbiased spatial distribution, the CoC value is 0, whereas positive CoC values indicate a rightward shift of space while negative CoC values indicate a leftward shift of space [18]. The following previously established cut-off values were used to define the presence of a neglect: Bells Test (CoC > 0.081 [13,18]), SNT (single version CoC > 0.081, dual version CoC > 0.118 [14]), Line Bisection Test (mean relative rightward deviation of >11% [15]) and Five-Point Test (CoC > 0.081, spatial distribution of graphic production [16,19]). In all three tests, the starting point was assessed (i.e., early orientation towards the left or the right [20]). 

Additionally, video-oculography during a free visual exploration paradigm (FVE) was performed in order to measure the spatial deployment of visual attention, as reflected by the horizontal distribution of visual fixations and early attentional orientation, i.e., the direction of the first saccade [17,20,21,22,23,24,25]. In brief, 12 naturalistic images (i.e., color photographs of everyday scenes; size 1200 × 900 pixels) and their mirrored versions (mirrored along the vertical axis) were shown on a computer screen. Each of the images was presented for 7 s and preceded by a central, black fixation-cross on a gray background (presented for 3 s), in order to enforce a common central starting point of visual exploration for all participants. The participants were motivated to freely explore the images, as if one would look at pictures in a photo album. A 3 × 3-point grid was presented for calibration of the eye-tracking system and for its validation prior to the experiment. During video-oculography, the participants were seated in front of the screen, and the head was positioned on a chin-and-forehead rest, to ensure that their mid-sagittal plane was aligned with the middle of the screen at a constant distance of 68 cm (resulting in a viewing angle of 28° × 21°) and to minimize head movements. Eye movements were recorded using a remote, infrared-based, video-eye-tracking system (EyeLink 1000 Plus System, SR Research, Ottawa, ON, Canada) [25].

FVE has been previously shown to be a very sensitive and reliable assessment tool to detect neglect after stroke [17]. The cut-off value for the mean gaze position during FVE is >1.333°, higher values indicating a pathological rightward shift of the distribution of visual fixations [17]. Early attentional orientation during FVE [17,20] was assessed by means of the landing point of the first fixation on each picture (left or right screen half), and the proportion of left- and rightward first fixations was computed. Early attentional orienting in healthy participants preferentially occurs towards the left hemifield [20]. To evaluate the patient’s performance, oculomotor data was statistically compared against those derived from 10 age-matched healthy controls [26].

To assess the severity of neglect in daily living, the Catherine Bergego Scale (CBS), a systematic and ecological observational questionnaire [27], was used. The established CBS cut-off ≥1 was considered as significant neglect (mild neglect 1–10, moderate neglect 11–20, severe neglect 21–30 [28]).

## 3. Results

The patient found all targets in the Bells Test (CoC = 0), but presented an early orientation towards the right space, i.e., began cancelling targets in the top right corner—a sensitive measure for neglect [20,30]. In the SNT, the patient showed a similar performance: both in the single task (CoC = −0.039) and in the dual task (CoC = 0.012), no spatial bias could be detected, but the patient started in the top right corner. In the Line Bisection Test, the patient performed normally (mean relative deviation of 1.30%). However, in a more demanding assessment—the Five-Point Test—the patient showed clear signs of left-sided neglect, i.e., the spatial distribution of graphical productions was clearly shifted towards the right (CoC = 0.338; Figure 1b).

**Figure 2:**
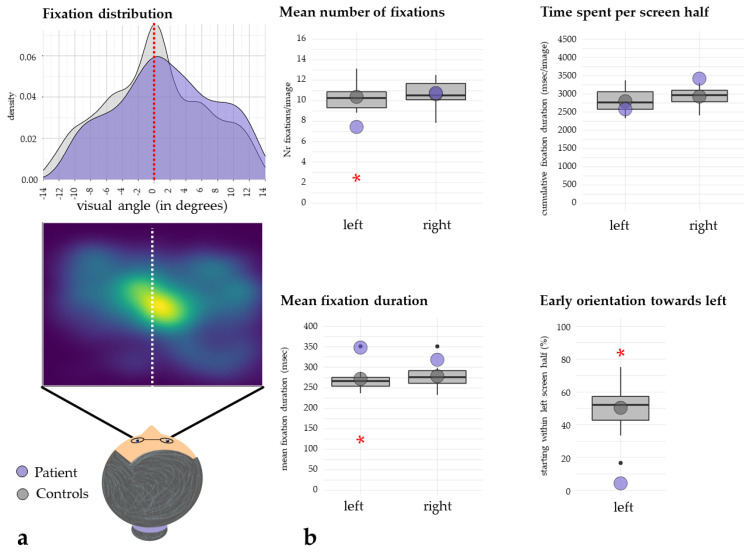
Oculomotor parameters. (**a**, **top**) A density plot of the fixation distribution in the patient (in violet) 24 days post-stroke and in 10 age–matched healthy controls (in grey); the red dotted line represents the middle of the screen. (**a**, **bottom**) A heatmap of the fixation distribution in the patient 24 days post–stroke; the white dotted line represents the middle of the screen. Both density and fixation plots show a shift in exploration behavior towards the right hemispace. (**b**, **top left**) Statistical comparison between the patient’s (violet dots) individual eye movements 24 days post–stroke and those of 10 age–matched healthy controls (grey dots, grey box plots), confirming a leftward neglect, with significantly less fixations within the left screen half. (**b**, **top right**) There was no significant difference between the patient and the healthy controls in time spent per screen half. (**b**, **bottom left**) The patient showed a significantly longer mean fixation duration on the left side. (**b**, **bottom right**) The patient directed significantly less first saccades towards the left hemispace compared to the healthy controls (* *p* < 0.05).

Furthermore, video-oculography during the FVE paradigm showed a typical pattern of left-sided neglect: the horizontal mean gaze position was significantly shifted towards the right hemispace (mean gaze position of 1.474°, which is above the cut-off of 1.333°, Figure 2a). Furthermore, statistical analyses ([26], Figure 2) comparing the patient’s visual fixation data against those derived from 10 age-matched healthy controls (no age difference, t = 0.714, *p* = 0.247), showed significantly fewer fixations within the left screen half (mean number of fixations per picture; patient: 7.42, healthy controls (HC): mean [SD] = 10.363 [1.34], t = −2.094, *p* = 0.033) but not in the right screen half (patient: 10.75, healthy controls (HC): mean [SD] = 10.642 [1.44], t = 0.072, *p* = 0.472; Figure 2b, top left). Compared to the healthy controls, the patient’s mean fixation duration was significantly longer in the left (patient: 347.83 ms, healthy controls (HC): mean [SD] = 271.83 [31.38] ms, t = 2.309, *p* = 0.023), but not in the right screen half (patient: 318.22 ms, healthy controls (HC): mean [SD] = 278.15 [33.65] ms, t = 1.135, *p* = 0.143; Figure 2b, bottom left). Interestingly, the time spent per screen half did not significantly differ between the patient and the control group for the left screen half (patient: 2579.7 ms, healthy controls (HC): mean [SD] = 2800.3 [342.36] ms, t = −0.614, *p* = 0.277), yet a trend was observable for the right screen half, i.e., the patient tended to spend more time within the right screen half, although this result was statistically borderline (patient: 3420 ms, healthy controls (HC): mean [SD] = 2926 [260] ms, t = 1.812, *p* = 0.052; Figure 2b, top right). Finally, first saccades during the FVE were significantly less often directed towards the ipsilateral, left hemispace (patient: 4.1667%, healthy controls (HC): mean [SD] = 50.31 [17.56]%, t = −2.505, *p* = 0.017; Figure 2b, bottom right). 

Additionally, the patient showed a mild neglect in the activities of daily living (CBS sum-score = 5, reflecting mild neglect [28]). The patient showed an impaired ability to look towards the left (CBS-item 5; score = 1; mild difficulty [28]) and to detect noise or voices from the left side (CBS-item 7; score = 1; mild difficulty [28]), collided with people and objects on her left (CBS-item 8; score = 1; mild difficulty [28]), and showed difficulties finding her way in familiar settings (CBS-item 9; score = 2; moderate difficulty [28]). 

Interestingly, in the MRI scan performed one day after stroke, the patient’s eye position revealed a significant deviation towards the contralateral side, supporting neglect diagnosis as described by Becker and Karnath [29] (Figure 1a). 

**Figure 3:**
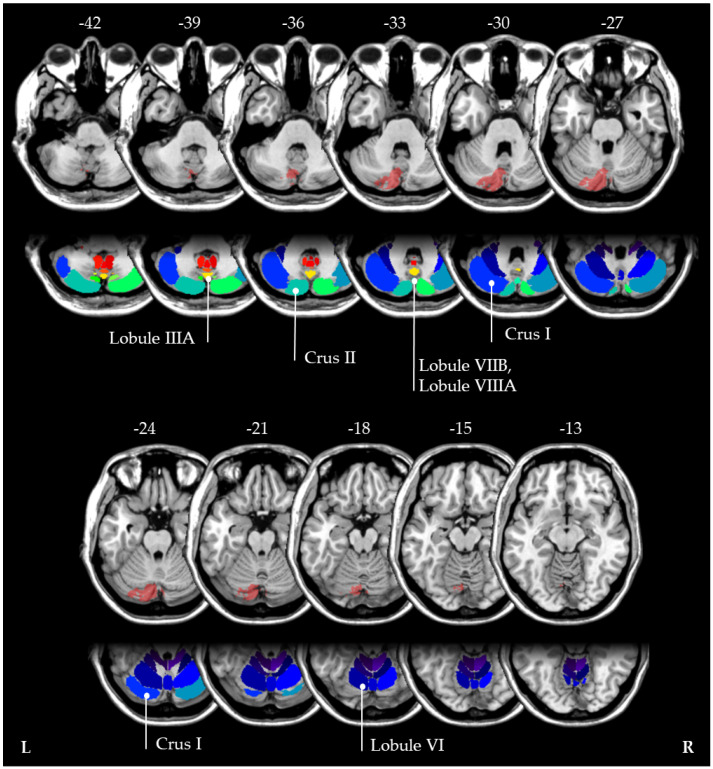
The MNI coordinates of the normalized cerebellar lesion (10 weeks post-stroke) are shown. Neuroanatomical localization of the patient’s lesion using FSL [33] revealed that the stroke affected crus I and II, as well as lobules IIIA, VI, VIIB and VIIIA.

To classify the neuroanatomical localization of the patient’s lesion, we used images from a second control MRI which was performed ten weeks after stroke. First, the patient’s individual cerebral lesion was manually delineated on individual structural MRI images using the open-source MRIcron software (https://www.nitrc.org/projects/mricron/, accessed on 17 February 2022). The borders of the lesion were manually delineated on every transverse slice of the individual MRI images. Then, images were normalized into MNI space with the Clinical Toolbox for SPM ([31]; https://www.nitrc.org/projects/clinicaltbx/, accessed on 17 February 2022), using enantio-morphic normalization [32], and running in SPM12 (http://www.fil.ion.ucl.ac.uk/spm, accessed on 17 February 2022). Furthermore, the “cerebellar atlas” that comes with FSL was used [33]. This revealed that the stroke affected crus I and II, as well as lobules IIIA, VI, VIIB and VIIIA (see Figure 3). Interestingly, the latter two lobules have been previously discussed as hubs for ipsilateral visuospatial cognition [34]. 

A follow-up assessment three weeks later revealed neglect remission in video-oculography during FVE (i.e., mean gaze position of −0.132°) and in daily living (CBS = 0), but not in the Five-Point Test (i.e., CoC = 0.137).

## 4. Discussion

The documented case shows the occurrence of ipsilateral visual neglect after an isolated stroke within the territory of the left PICA. These results show that the posterior and inferior part of the cerebellum participates in the control of ipsilateral attention, particularly highlighting the importance of the cerebellum in spatial attention allocation. 

One day after stroke, the patient showed a rightward eye deviation in the MRI scan, a clear sign of neglect (Figure 1a; [29]). In the neurorehabilitation unit, twenty-four days post-stroke, the neglect, however, was mild and only detectable when using the sensitive neuropsychological Five-Point Test as well as video-oculography assessment. The patient showed a significant shift in the visual fixation distribution towards the right hemispace (Figure 2a). Furthermore, the first saccade was significantly more often directed towards the right hemispace (Figure 2b).

However, the neglect remained unnoticed when evaluated with common neuropsychological paper-pencil tests. Furthermore, three weeks later, the follow-up assessment revealed a net amelioration of neglect symptoms. Therefore, these findings suggest that visual neglect may be a part of CCAS, but that the choice of neglect assessments and the time delay since stroke onset may be crucial. 

The findings regarding neglect after cerebellar lesions are ambiguous. Cerebellar neglect was previously described in sparse single-case studies. Silveri et al. [9] documented the case of a patient with an ipsilateral, right-sided hemineglect after a right cerebellar haemorrhage extending to the vermis. Interestingly, another case reported by Hildebrandt et al. [8] showed a contralateral, left-sided neglect after a right PICA stroke. However, the exact extent and location of the lesion in this patient could not be determined because of a clip artefact in the MRI scan. 

Another study by Kim et al. [35] found mixed evidence. They conducted a study with a higher number of patients, i.e., 28 patients with cerebellar stroke. A total of 8 out of the 28 assessed patients were diagnosed with neglect. In these eight patients, four patients had a left cerebellar stroke (three showing contralateral neglect and one ipsilateral neglect) and four patients had a right cerebellar stroke (three showing ipsilateral neglect and one contralateral neglect). However, the lesions were not restricted to the PICA territory, and the one patient with an isolated left PICA stroke did not show any signs of neglect. Furthermore, lesions affecting the vermis led to neglect more often than lesions in the cerebellar hemispheres, a finding which is congruent with the observations in our patient, whose lesion also involved the vermis. 

Other studies concluded that neglect does not occur after cerebellar stroke. In a study by Baier et al. [10], 16 patients with unilateral cerebellar lesions were assessed for neglect in the acute post-stroke stage. Only 1 out of these 16 patients showed neglect, which was contralateral after a right cerebellar lesion. Frank et al. [11] investigated 22 patients with acute unilateral left or right cerebellar stroke and compared the results of this group with those of a healthy control group. On the group level, no statistical difference in neglect tests was observed between the two groups. Qualitatively however, neglect patients with a right cerebellar lesion showed worse performance than patients with a left cerebellar lesion, and the control group. The authors concluded that neglect does not typically occur after cerebellar strokes. These findings are congruent with the ones by Richter et al. [12], who performed a study in 14 patients with a PICA infarct and seven patients with an SCA infarct. While none of the patients showed visual neglect in the chronic post-stroke stage, a non-significant trend of rightward deviation in the Line Bisection Test was observable in patients with left cerebellar stroke. 

One possible reason for this discrepancy in findings and interpretations regarding the contribution of the cerebellum in the spatial allocation of attention may concern the choice of assessment methods. First, neglect might remain undetected when using standard neuropsychological paper-pencil tests that are not sensitive enough to diagnose mild forms of the disorder [17]. Indeed, the above-mentioned previous studies [10,11,35] assessed the presence of neglect by means of the Line Bisection Test [36], different cancellation tasks [37,38,39], or different figure copying tasks [40,41]; these tests have been shown to have a neglect detection rate up to 50% [17]. Correspondingly, the presence of neglect in our patient would not have been detected if only the Line Bisection Test [15] or different cancellation tests [13,14] had been administered. However, the presence of neglect was evident when considering a more sensitive parameter, i.e., the spatial distribution of graphic productions in the Five-Point Test [16,19]. Furthermore, neglect was observable during FVE. The FVE paradigm is a more accurate and sensitive neglect screening tool than conventional paper-pencil tests, and is even more sensitive than a combination of several of such conventional assessments [17]. As the studies by Richter et al. [12] and Frank et al. [11] evidenced neglect-like trends by means of conventional paper-pencil assessments, it is therefore possible that more sensitive assessment tools would have detected and reflected neglect more clearly. 

Our report of cerebellar neglect shows parallels with the descriptions of language deficits after cerebellar strokes. After right-hemispheric cerebellar strokes, only mild linguistic difficulties may occur, such as modestly impaired semantic access, grammatical deficits and impaired rhythm and prosody [42,43]. In this case, impairments in these more complex linguistic functions may remain undetected if only standard language assessments are used [42]. Hence, analogue to the discussion about the occurrence of neglect after cerebellar stroke, the occurrence of the so-called cerebellar-induced aphasia [44] remains controversially debated.

A second reason for the discrepancies in reports of neglect after cerebellar stroke might be based on plasticity during the recovery process, which may in turn influence neuropsychological test results. Indeed, in our patient, the neglect after PICA stroke improved quickly within the subacute phase, as shown in the follow-up assessment three weeks after the initial examination. Therefore, studies conducted in patients with chronic cerebellar stroke (e.g., Richter et al. [12]) may come to different results because these plastic changes may already have taken place. 

Third, the heterogenous results regarding ipsi- and contralateral neglect might have anatomical reasons. For instance, in most of the above-mentioned previous studies, it was not clearly stated whether the strokes were purely isolated to the cerebellum, or whether additional brainstem structures were also affected. Theoretically, it is possible that additional afferent or efferent cerebellar fibres might also be damaged [7], which could explain the reported discrepancies between ipsi- and contralateral neglect occurrence. In this context, it is interesting to note that in our patient, the stroke affected the left lobules VIIB and VIIIA. These regions have recently been discussed as hubs that code key aspects of ipsilateral visuospatial representations [34].

In summary, the present findings suggest that not only executive, language, and affective deficits may occur within the framework of the Schmahmann Syndrome [6], but also, particularly if lobules VIIB and VIIIA [34] are affected, visual neglect. Furthermore, these findings underline the view that the cerebellum has a modulatory role on cortical function [4]. Neuroanatomical and physiological results evidenced cortico–cerebellar circuits connecting the cerebellum to the contralateral association cortex [1,7]. In particular, in case of a PICA stroke, the ventral posterior cerebellum might become disconnected from contralateral parietal and associative areas [45]. It seems therefore plausible that a PICA stroke might result in hypoactivation and diaschisis within the corresponding contralateral telencephalic hemispheric cortical networks [3], leading to neglect after a left-hemispheric PICA stroke and to aphasia after a right-hemispheric PICA stroke. 

A PICA stroke often results in a CCAS and—in our patient—in neglect. It would be interesting to further investigate whether neglect only occurs after lesions of lobule VIIB and VIIIA or whether other cerebellar structures are also involved. To determine the incidence of neglect in patients with cerebellar strokes, a prospective assessment in a large number of these patients would be needed. An assessment by means of highly sensitive tools, both in the acute and subacute stage of recovery, would offer the exciting possibility to further elucidate the pathophysiological mechanisms of cerebellar contribution to visual attention allocation.

## Data Availability

The conditions of our ethics approval do not permit public archiving of the data supporting the conclusion of this study. Readers seeking access to the data and study materials should contact the lead author Thomas Nyffeler. Requestors must complete a formal data sharing agreement to obtain the data. All data that are necessary and sufficient to replicate all data processing steps and analyses will be shared to requestors who meet these requirements. Access to the data and study materials will be permitted as part of a collaboration upon a case-by-case decision.
